# Evolutionary Footprints of Short Tandem Repeats in Avian Promoters

**DOI:** 10.1038/srep19421

**Published:** 2016-01-14

**Authors:** Hideaki Abe, Neil J. Gemmell

**Affiliations:** 1 Department of Anatomy, University of Otago, Dunedin 9054, New Zealand; 2 Allan Wilson Centre for Molecular Ecology and Evolution, University of Otago, Dunedin 9054, New Zealand

## Abstract

Short tandem repeats (STRs) or microsatellites are well-known sequence elements that may change the spacing between transcription factor binding sites (TFBSs) in promoter regions by expansion or contraction of repetitive units. Some of these mutations have the potential to contribute to phenotypic diversity by altering patterns of gene expression. To explore how repetitive sequence motifs within promoters have evolved in avian lineages under mutation-selection balance, more than 400 evolutionary conserved STRs (ecSTRs) were identified in this study by comparing the 2 kb upstream promoter sequences of chicken against those of other birds (turkey, duck, zebra finch, and flycatcher). The rate of conservation was significantly higher in AG dinucleotide repeats than in AC or AT repeats, with the expansion of AG motifs being noticeably constrained in passerines. Analysis of the relative distance between ecSTRs and TFBSs revealed a significantly higher rate of conserved TFBSs in the vicinity of ecSTRs in both chicken-duck and chicken-passerine comparisons. Our comparative study provides a novel insight into which intrinsic factors have influenced the degree of constraint on repeat expansion/contraction during avian promoter evolution.

Promoters are genomic *cis*-elements, generally located in the upstream regions of genes. It is now well established that eukaryotic promoters contain various types of short sequence motifs that might directly or indirectly influence transcriptional regulation by modifying interactions with the polymerase complex^1^. For example, transcription factor binding sites (TFBSs) are short binding sequences for transcription factor (TF) proteins and preferentially cluster near the transcription start site (TSS) of most genes^2^. Short tandem repeats (STRs) are also found ubiquitously in the promoters of both higher and lower eukaryotes^3^,^4^. The disruption of TFBSs undoubtedly has a critical impact on transcriptional activity; however, previous experimental studies have provided evidence that insertions or deletions (indels) may affect gene expression by changing the number of TFBSs (i.e. tandemly repeated TFBSs)^5^,^6^ or alter the spacing between different regulatory sites^7^. These empirical observations, together with the previous co-occurrence approach of binding sites^8^,^9^, suggest that spacing between functional TFBSs is critically important for the optimal formation of a nucleoprotein structure in regulatory regions.

To date, cross-species comparisons of TFBSs indicate that selection, as well as mutation/random genetic drift, determine the evolutionary fate of TFBSs^10^,^11^. Assuming that the expansion or contraction of some STRs may occasionally affect the position and distance specificity of functional TFBSs, such mutations on STR loci should be under strong constraint at the equivalent level as those acting on the nearest TFBSs. Indeed, Cameron *et al.* showed that indels are suppressed inside *cis*-regulatory modules, relative to their neighboring promoter sequences^12^. Other researchers also found a lower density of short indels in the upstream regulatory regions (−100 ~ 0 bp) than in other regions (e.g. introns, both untranslated regions) of chicken protein-coding genes^13^. Therefore, it seems likely that strong selection against indels and/or stabilizing selection has acted on the number of repeat units, allowing them to maintain repeat characteristics within populations, species, and more diverged taxa.

Despite their structural and evolutionary significance, only a few studies have examined the polymorphisms of STR loci embedded in orthologous promoter regions across diverged vertebrates. Ohdai and co-workers have shown that evolutionary conserved STRs (ecSTRs) are sporadically found in the core promoter regions, not only among major primates but also between primate and non-primate vertebrates^14^. Similar findings have been reported by Sawaya *et al.*, who show that promoter region of the *Nos1* contains ecSTRs among major vertebrate clades, each with a distinct number of AC/GT units^15^. Although these data hint at the important role of ecSTRs in transcriptional regulation, the underlying evolutionary trajectory has yet to be elucidated.

In our previous study on sequence motifs in the chicken promoter, we showed that STR motif frequencies in promoter regions are evolutionary conserved across vertebrates (human, mouse, chicken, duck, and zebra finch), even though total abundance and expansion patterns of STRs largely differ between avian and mammalian promoters^3^. These outcomes simply indicate that motif biases specific to the upstream promoter region are conserved to some extent, whereas the overall pattern of expansion/contraction of STRs is constrained in a lineage-specific manner. To improve our understanding about the evolutionary trajectory of STRs in vertebrate promoters, in this study, we identified ecSTR loci in orthologous promoter sequences from five avian species (chicken, turkey, duck, zebra finch, and flycatcher) and carefully examined the intrinsic characteristics of ecSTRs, including conservation rates, types of repeat motifs, and relative distance from TFBSs. Our cross-species comparisons were made between chicken and the four other birds to highlight the variety of avian phylogenetic distances from low (chicken–turkey), moderate (chicken–duck), and high [chicken–passerines (zebra finch/flycatcher)] divergence. The present study offers an intriguing opportunity to trace the footprints of ecSTRs in avian promoters and emphasizes the important role of repetitive sequence motifs as components of promoter architecture.

## Results

### Abundance of short tandem repeats in avian promoters

Initially, we investigated the characteristics of avian promoters and abundance of sequence motifs (Table 1). The total number of STRs was lowest in the turkey (perfect: 973; imperfect: 2,122) and highest in the zebra finch (perfect: 1,631; imperfect: 3,784). The high interspecific variation in the number of sequence motifs might be due to differences in the quality of genome assembly or in strategic options for an assembly, otherwise it simply reflects underlying biological differences. In all avian species, except turkey, tetranucleotide or longer STRs had larger numbers of repeat units than di- and trinucleotide STRs (Kruskal-Wallis test; *p* < 0.05). A comparison of the frequencies of STR motifs showed motif biases (Supplementary Figure S1) and a significant difference in motif usage between two divergent avian groups, Galloanserae [Galliformes (chicken/turkey) + Anseriformes (duck)] and Passeriformes (zebra finch/flycatcher). A pairwise comparison of STR motif frequencies made between avian species showed that 11 out of 60 comparisons between Galloanserae and Passeriformes were significantly different at the 1% level (F-test: *p* < 0.01), whereas three out of 40 were significant within each group (Supplementary Figure S2). Thus, different levels of evolutionary constraints on STR motif frequencies were statistically significant between Galloanserae and Passeriformes (Fisher’s exact test; *p* < 0.001).

### Short tandem repeats are shared across closely and distantly related avian species in their promoters

We found considerable differences in the ratio of conserved STRs between the comparisons of chicken–turkey and chicken–other birds (Supplementary Figure S3). The total abundance of ecSTRs largely differed between the turkey and other birds (duck, zebra finch, and flycatcher), with more than half (52.2%) of the ecSTRs being identified in the comparison between chicken and turkey. The number of ecSTRs that met the screening criteria was 149 perfect repeats [turkey: 78 (8.0%); duck: 27 (2.0%); zebra finch: 29 (1.8%); flycatcher: 15 (1.1%)] and 267 imperfect repeats [turkey: 139 (6.6%); duck: 50 (2.0%); zebra finch: 47 (1.2%); flycatcher: 31 (1.3%)]. This finding is consistent with other studies showing that chicken and turkey are phylogenetically close relatives whose overall genome architectures are quite similar^16^,^17^, even though they diverged 20 ~ 40 million years ago (MYA)^18^.

### Conservation rates differ among repetitive motifs

**Figure 1:**
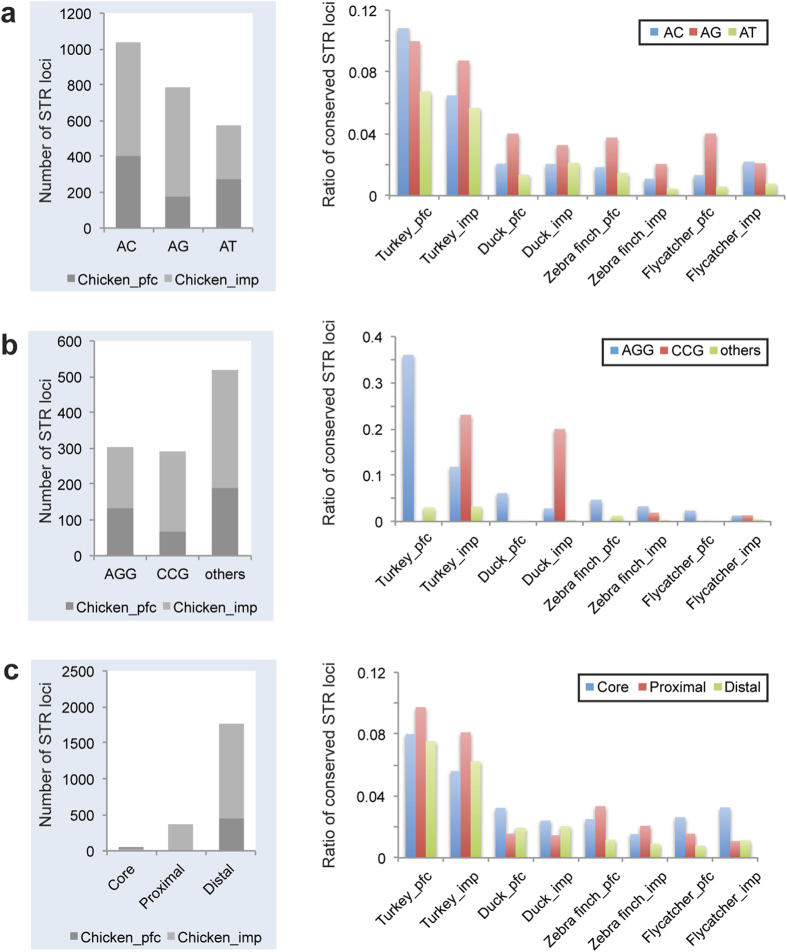
The ratio of conserved STR loci among dinucleotide repeats, trinucleotide repeats, and promoter regions. The ratio of evolutionary conserved STRs (ecSTRs) is shown for dinucleotide repeats (**a**), trinucleotide repeats (**b**), and promoter regions (**c**). Data from CG repeats were excluded due to the extremely small sample size, while only dominant motifs were compared among trinucleotide repeats. The actual number of STR loci detected in the chicken promoter is shown in the left on each panel for a comparison. In panel C, the promoter sequence is divided into three parts: core (−1 ~ −100 bp), proximal (−101 ~ −500 bp), and distal (−501 ~ −2000 bp). Abbreviations used in this figure are pfc [perfect (pure) repeat], imp [imperfect (interrupted) repeat].

The vast majority of ecSTRs among avian promoters were found to contain di- or trinucleotide repeats, thereby ecSTRs with tetra- or longer repeats (less than 1% of total ecSTRs) were excluded from the following analyses. A more detailed analysis revealed that conservation rates were also largely different among the types of dinucleotide motifs (Fig. 1a). The proportion of AG motifs conserved was higher than that for other dinucleotide motifs (Kruskal-Wallis test; *p* < 0.05). In trinucleotide STRs, AGG and CCG were predominant motifs that were conserved in Galloanserae, whereas trinucleotide loci were rarely conserved in Passeriformes (Fig. 1b). We also found that ecSTRs were predominantly detected in either the proximal regions (turkey/zebra finch) or in the core regions (duck/flycatcher) of promoters (Kruskal-Wallis test; *p* < 0.05; Fig. 1c).

**Figure 2:**
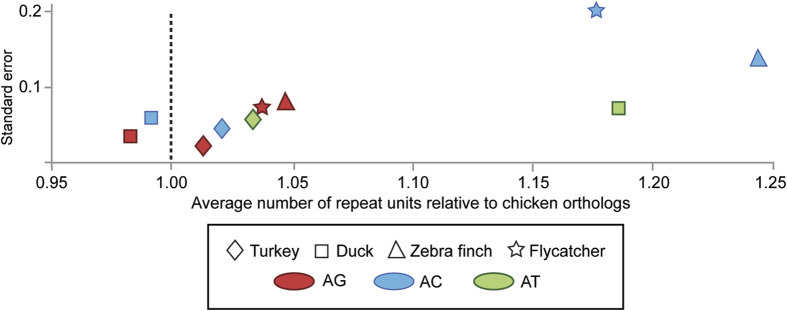
Average number of dinucleotide repeat units relative to chicken orthologs in evolutionary conserved STR loci. The average number of repeat units was compared among dinucleotide repeats. Data from CG and AT STR motifs in passerines (zebra finch and flycatcher) were eliminated due to insufficient data. Vertical dotted line indicates the standardized number of repeat unit in chicken STRs as 1.00.

There was no obvious trend in the scatter plots for the number of repeat units between chicken and other bird species (Supplementary Figure S4). In turkey and duck, however, the average number of units relative to the chicken orthologs suggested more constraint on AG motifs than either AC or AT (Fig. 2 and Supplementary Figure S5). These differences between AG and AC motifs were more prominent in passerines than in Galloanserae (the abundance of STRs with AT motifs was insufficient to allow comparisons in passerines). The expansion and contraction of AG motifs appears to be significantly suppressed in the turkey (χ^2^ test: *p* < 0.05; Table 2). In contrast, there was no discernible pattern or trend in ecSTR unit expansion/contraction when promoter sequences were divided into distal, proximal, and core regions.

### Evolutionary direction of repeat expansion or contraction

**Figure 3:**
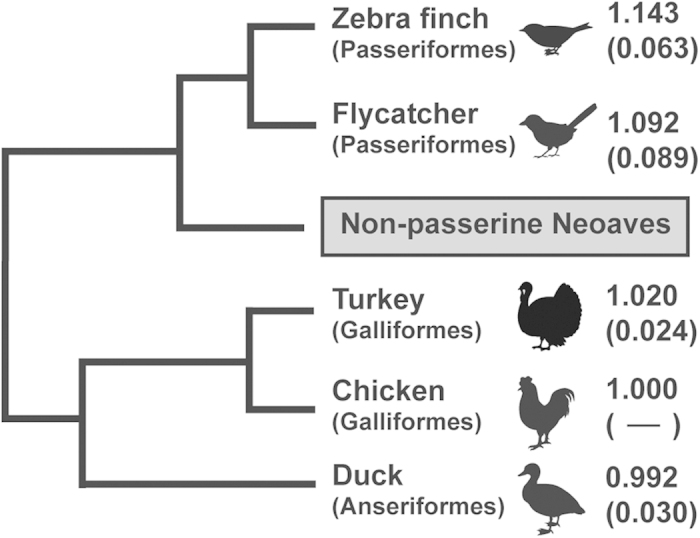
Avian clade and the average number of repeat units relative to the chicken orthologs. The average number of repeat units was calculated in the evolutionary conserved STR loci with dinucleotide motifs. Branch lengths do not precisely correspond to the divergent time estimated in the previous study^25^. Standard errors are shown in parentheses. Neoaves is the large avian group, including most of the extant birds, except Galliformes (e.g. chicken, turkey), Anseriformes (e.g. duck), and Ratitae (e.g. ostrich).

**Figure 4:**
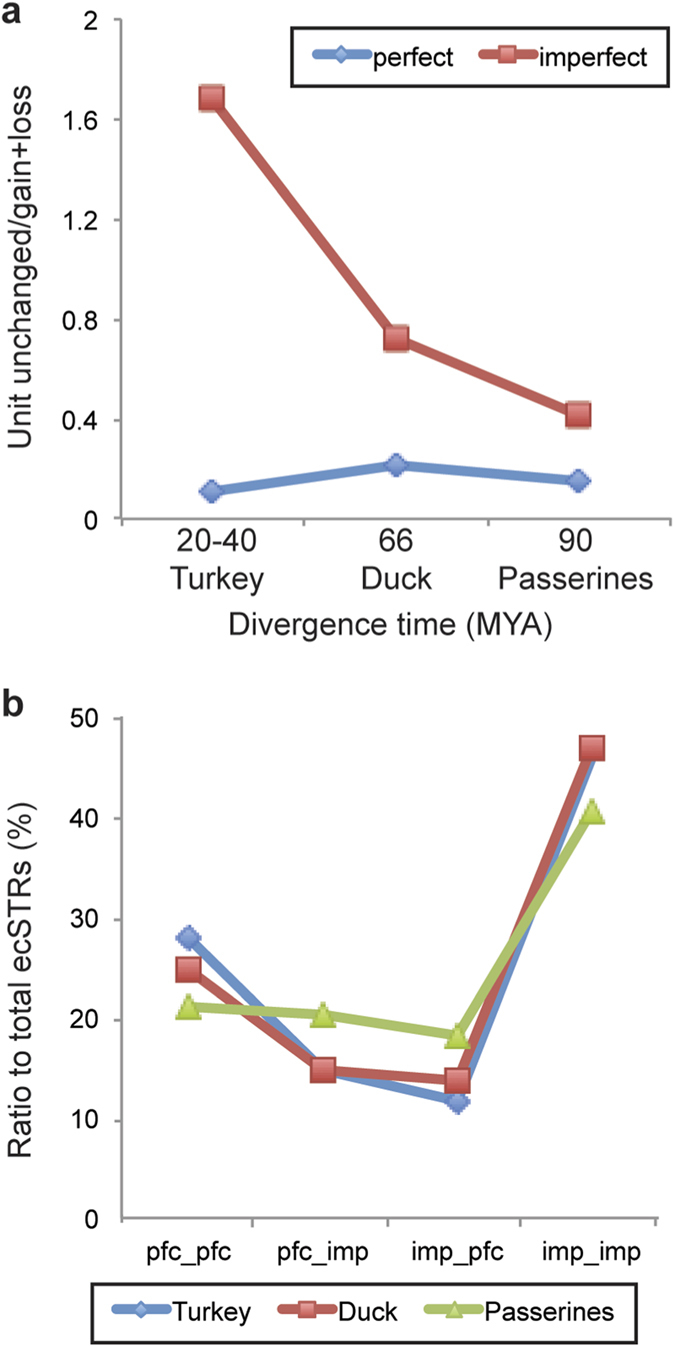
Evolutionary trends of the conserved short tandem repeats with perfect or imperfect dinucleotide repeats. The Y-axis represents the rate of evolutionary conserved STR (ecSTR) loci that have the same number of repeat units with chicken orthologs against those with gain/loss of units. The X-axis denotes the estimated divergence times from the chicken lineage (**a**). EcSTRs were sorted into four categories based on their repeat purity, and compared in ratio among avian lineages (**b**). Here, we express the repeat purity of ecSTRs as “chicken repeats_ortholog repeats”. For example, *pfc_pfc* means that both orthologs have ecSTRs with perfect repeats, with the same being true for imperfect repeats (*imp_imp*). Note that several ecSTRs (13.9% to total ecSTR loci) were double counted across different categories as distinct types of repeats. Abbreviations used in this figure are pfc [perfect (pure) repeat], imp [imperfect (interrupted) repeat].

Figure 3 shows the grand average of repeat unit numbers with the standard error for each avian species to chicken. In Galloanserae, the turkey had slightly longer repeats against chicken orthologs, whereas the duck had slightly shorter repeats. Both passerines were characterized with a larger number of STR units than Galloanserae. The gain/loss rate trends of repeat units largely differed between avian lineages as well as between perfect and imperfect repeats. When compared to chicken STRs, there was significant difference in gain/loss rate between repeat purities among bird taxa (Fig. 4a; Kruskal-Wallis test; *p* < 0.05), whereas there was neither significant difference in the duck nor in passerines. When we categorized ecSTR loci with dinucleotide motifs into four directional shifts in their repeat purities (perfect to perfect, perfect to imperfect, imperfect to perfect, and imperfect to imperfect), no significant difference in their ratio was observed between avian lineages (Fig. 4b).

### Abundance of conserved TFBSs within the avian promoters

A total of 18,737 TFBSs were identified in 163 promoter sequences that contain ecSTRs (114.95 TFBSs per promoter sequence) in the chicken–turkey comparison. Meanwhile, 2,512 TFBSs were detected in 74 sequences conserved between the chicken and duck (33.95 per sequence) and 2,216 TFBSs were detected in 86 sequences conserved between the chicken and passerines (25.77 per sequence). When we sorted TFBSs into 100 bp bins, excess rates of FREAC-7 (Forkhead-Related Transcription Factor 7) were detected in the vicinity of ecSTRs in all comparisons (Kruskal-Wallis test; *p* < 0.01; Supplementary Figure S6). The FREAC-7 was expected to target the ATAAA[C/T]A motif^19^ and thus overestimated the number of STRs with imperfect AT motifs that co-occurred with FREAC-7. Therefore, we deleted FREAC-7 data counted within 20 bp from the first nucleotide of the ecSTRs with AT motifs.

### Relative distances estimated between ecSTRs and TFBSs

**Figure 5:**
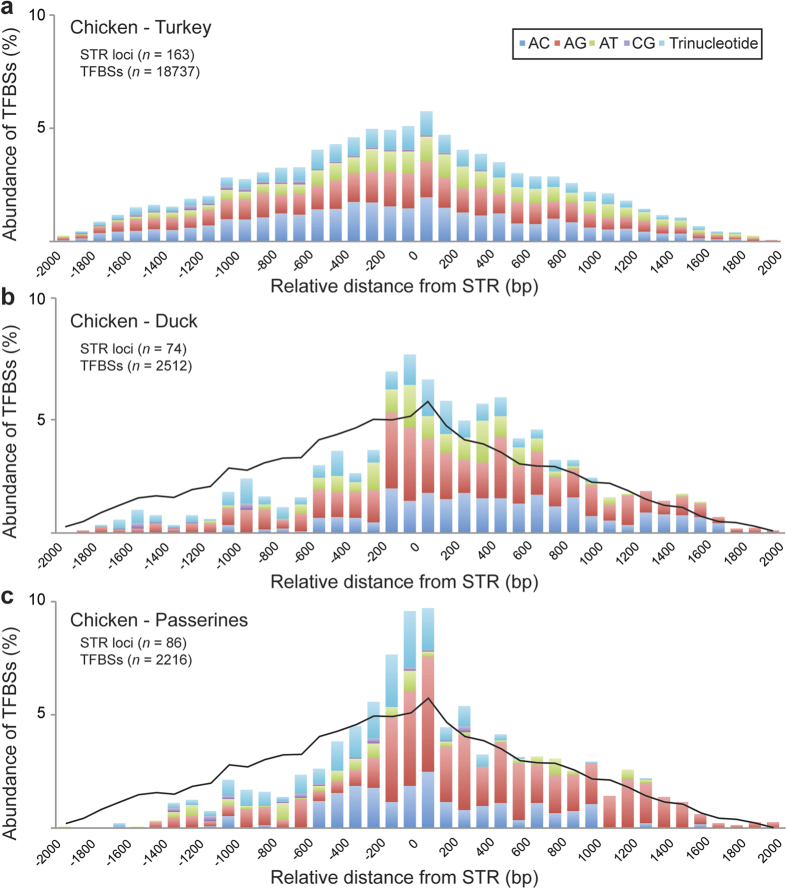
Relative distances between evolutionary conserved short tandem repeats (ecSTRs) and transcription factor binding sites (TFBSs). Relative distances between conserved sequence motifs were calculated in chicken–turkey (**a**), chicken–duck (**b**), and chicken–passerines (zebra finch/flycatcher) comparisons (**c**). Only di- and trinucleotide repeats were counted here in a 100 bp bin. Here we used position data from the chicken promoter sequences. Each peak in panel **a** was traced in panels (**b**,**c**), allowing for easy comparison. Note that the rate of conserved TFBSs is noticeably higher downstream of ecSTRs in panels (**b**,**c**), mainly due to the biased distributions of TFBSs near transcription start sites.

**Figure 6:**
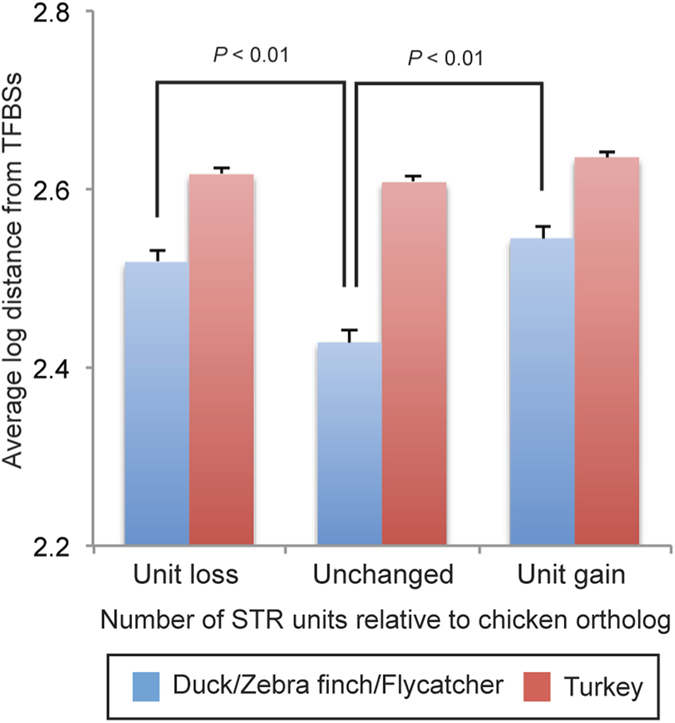
Average log distance between TFBSs and ecSTRs with different trends of unit expansion. The cohorts “Unit gain” and “Unit loss” indicate the gain and loss of repeat units relative to chicken orthologs, respectively. “Unchanged” means that exactly the same number of repetitive units is found in orthologous repeat loci. Comparisons are made either between chicken and their close relative (turkey; depicted in red bars) or between chicken and diverged avian groups (duck/zebra finch/flycatcher; depicted in blue bars).

In the chicken–turkey comparison, the conserved TFBSs were normally distributed in relation to the relative distance from ecSTRs (Fig. 5a). In contrast, skewed distributions were obtained in the chicken–duck and chicken–passerine comparisons (Fig. 5b,c). The excess accumulation of conserved TFBSs near ecSTRs was more prominent in chicken–passerines compared to chicken–turkey. When we considered the distributions of ecSTRs with AG and AC motifs separately, we detected biased distributions of conserved TFBSs between motifs in chicken–duck (Kruskal-Wallis test; *p* < 0.05) and chicken–passerine (*p* < 0.05) comparisons, but there was no significant difference in chicken–turkey. The average log distance between ecSTRs and conserved TFBSs was almost constant, regardless of the number of repeat units in the chicken–turkey comparison. However, the average distance between each pair of ecSTR and TFBS was significantly smaller in ecSTRs that exhibited a stable (unchanged) number of repeat units between the chicken and the other species (duck/zebra finch/flycatcher) based on the two-tailed t-test (unit loss vs. unchanged, *p* < 0.001; unit gain vs. unit unchanged, *p* < 0.001; unit loss vs. unit gain, n.s.; Fig. 6).

## Discussion

Promoters have a complex architecture, containing a variety of short sequence motifs such as STRs, G-quadruplex (G4), and TFBSs. An increasing number of these motifs have known functions, but the roles of others remain less clear. As we show here, investigations of the evolutionary conservation and dynamics of these sequence motifs among closely and distantly related species as well as the genomic context in which they are found may provide important clues that enable us to further identify those that are functionally important, from those are not.

In this study we cannot exclude the possibility that intra-specific variation in a certain number of ecSTRs might affect the major analytical results. Our pilot study found that a few ecSTR loci (1/10 tested) in promoter regions showed intra-breed variation in a particular breed of chicken (data not shown). Technically, the level of intra-specific polymorphisms in promoter STRs would be more efficiently estimated by using next generation sequencing data in the near future. At this point in time, however, it is quite difficult to precisely assess the frequency of intra-specific STR loci in a particular species, because the ratio of STR loci that show intra-specific polymorphism would vary depending either on the number of analyzed individuals or the relatedness of sampling colonies. Therefore, we do not mention intra-specific variation in this paper to avoid confusion on the definition of ecSTRs.

Several hypotheses can be made to explain the difference in STR motif abundance in avian promoters. The skewed motif distribution of ecSTRs might be derived from the different degree of slippage mutability for dinucleotide motifs. Kelkar *et al.* illustrated that four types of dinucleotide repetitive motifs differ in their mutability patterns^20^. For orthologous STR loci compared between human and chimpanzee, mutability was significantly higher for AC than AG and AT motifs at low repeat numbers (6 ≤ *n* < 11), whereas this pattern was reversed in longer repeats (*n* ≥ 11). Of note, the rank of STR mutability at low repeat numbers (AT > AC  AG) is in good accordance with the rates of ecSTRs identified between chicken and the orthologs of the other four bird species (AT < AC  AG) evaluated in this study (Fig. 1a). Therefore, a low level of mutability in AG repeats may help to maintain several intrinsic features of repeat sequences, including motif size, length (number of units), sequence composition, and repeat purity, throughout avian promoter evolution. An alternative, but not mutually exclusive mechanism contributing to the higher rate of AG repeats in ecSTRs, may be associated with distinct levels of selective pressure acting on dinucleotide motifs. It is widely known that, along with the trinucleotides GAA or AGG motifs, AG dinucleotide sequences may adopt a non-B DNA structure, an intramolecular triplex, or H-DNA^21^. Indeed, AG motifs, identified in the human core promoters of *SOX5*, *MECOM*, and *GABRA3*, are highly conserved across divergent mammals^22^, thus suggesting that purifying selection is considered as another primary force shaping an elevated ratio of AG repeats in ecSTRs.

Several papers have estimated the ratio of mutational bias toward the expansion or contraction of dinucleotide repeats in vertebrate genomes^23^,^24^. Some of these papers clearly showed that AC motifs are the major contributors for STR expansion, whereas the average number of repeat units appears to be fewer in STRs with AG motifs. Our data on the average number of ecSTR units were in agreement with these previous findings. When comparing four birds against the chicken orthologs, the average number of AC repeat units was always higher than that of AG motifs. However, the extent of AC motif expansion largely differed between Galloanserae (turkey/duck) and Passeriformes (zebra finch/flycatcher). A marginally longer AC motif was detected in the comparison between the chicken and other Galloanserae, whereas this difference was very large in Passeriformes (Fig. 2). Given that the divergence time between Galloanserae and Neoaves lineages would be around 70 MYA^25^, a much higher level of constraint on the AC motif between the duck and chicken lineages is likely to be inconsistent with molecular phylogeny of birds indicating that Anseriformes (duck) separated from Galliformes (chicken/turkey) around 46 MYA^25^.

One possible scenario for this discrepancy is the different rate of molecular evolution between Galloanserae and Passeriformes. Nam *et al.* recently suggested that there is a difference in the molecular clock of these two parallel lineages^26^. The zebra finch lineage had a significantly higher overall nucleotide substitution rate (ω) in protein coding sequences than the chicken lineage, leading to the conclusion that passerine genes and maybe their genomes evolve more rapidly than non-passerines. A similar outcome was obtained in a comparative analysis of duplicated growth hormone genes among avian lineages^27^. The authors concluded that the higher evolution rate of this gene in passerines is attributed to differential selection, with possible implications for the explosive diversification of the passerine clade. These studies suggest that key aspects of the biology of passerines, such as the short generation time and large effective population size, may have critically affected the overall evolutionary rates of STR expansion. Indeed, computation simulations suggested that fluctuations in population size could be an influencing factor on mean repeat size differences in the process of STR maturation^28^.

The purity of repeat motifs is another intrinsic factor that influences the conservation rates of repeat units. In this study, conservation rates in repeat units exhibited a distinct trend between ecSTRs containing either perfect or imperfect repeats. In the turkey, the ecSTR loci with imperfect repeats had a significantly higher number of “unchanged” repeat units with chicken (Fig. 4a). The simplest explanation is that several nucleotide substitutions in repetitive motifs would precede the slippage dinucleotide mutation particularly between recently diverged groups. These interruption mutations have a major impact on subsequent STR expansion, by affecting the rate of slippage^29^. The patterns of repeat purity were strictly conserved in Galloanserae, with a different trend being observed between the chicken and passerines. In the chicken–duck comparison, for example, approximately 30% of repeat motifs underwent a shift in repeat purity either in the chicken (indicated as *imp_pfc* in Fig. 4b) or in the duck (*pfc_imp*) ecSTR loci. This ratio is significantly higher in the passerine lineage, suggesting that not only stepwise mutation but also the shift of repeat purity contributed to avian promoter divergence.

Several previous studies have shown that *cis*-regulatory motifs are positionally constrained within 100 bp upstream of the TSS^2^,^8^,^30^. More specifically, if two TFs interact with each other, their TFBSs in the promoter do not show a random distribution but, rather, tend to be close each other^31^,^32^. Because an expansion or contraction of STRs in the promoters inevitably alters the relative distance between functional TFBSs, positional constraints might also act on ecSTRs to maintain the optimal distance between TFBSs. The patterns of relative distances between ecSTRs and JASPER TFBSs are largely different among avian species. The turkey, which is the closest relative to the chicken, showed a normal symmetric distribution of conserved TFBSs in conjunction with ecSTRs (Fig. 5a). In contrast, the duck and passerines had a significantly higher rate of conserved TFBSs in the vicinity of ecSTRs (Fig. 5b,c). These distinct patterns of distribution may simply reflect selection bias over evolutionary time since these species split from the chicken lineage. The turkey and chicken have a similar genomic composition to each other, regardless of their deep divergent splits (around 20 ~ 40 MYA). Thus, weak selective pressure may have acted on the distance between regulatory motifs. In other words, the current level of motif divergence between the chicken and turkey is far from saturation under purifying selection. However, the rate of conserved TFBSs in passerines appears to be higher in the vicinity of ecSTRs, which is probably due to strong selection pressure acting on distantly located motifs in the promoters. These results are consistent with a previous report stating that location-specific motifs have been subject to evolutionary changes in the consensus sequence based on the divergence times between organisms^33^.

Another interesting finding is that the ecSTRs with AG repeat motifs had a significantly higher proportion of conserved TFBSs than those with AC motifs in both the chicken–duck and chicken–passerines comparisons (Fig. 5). This phenomenon cannot be explained by the different rates of slippage mutability among dinucleotide repeats or by the higher expansion rate observed in AC motifs. A recent finding on the transcriptional importance of non-B DNA structures in *Escherichia coli* provides a pivotal clue about biased distribution of AG motifs near TFBSs^34^. H-DNA motifs appear to be enriched in the regulatory regions of transcriptional units, suggesting the critical importance of the helical phase of short DNA motifs in specific regulatory regions^21^. This hypothesis provides a plausible explanation for our observation, on the grounds that synergistic transcription activation is experimentally mediated by the distance between two *cis*-regulatory elements (i.e. distance dependence), as well as their stereospecific positioning (i.e. helical phase dependence)^35^.

Finally, we detected a significant difference in the average distance between TFBSs and ecSTRs, depending on the stability in the number of repeat units. The mean distance between ecSTRs and conserved TFBSs was significantly constrained only when the number of repeat units was “unchanged” between chicken and three of the divergent bird species (duck/zebra finch/flycatcher), whereas this trend was not observed in the chicken–turkey comparison (Fig. 6). These results support our hypothesis that the expansion and contraction of STR units has been under strong constraints during avian evolution, especially when ecSTRs are located near TFBSs.

Here we identified a large number of promoter STRs that are conserved between diverged avian species, and examined the factors that might explain the level of evolutionary constraint. Our findings suggest that selective pressure has not uniformly acted on repeat motifs, but that AG repeats have been subject to severe constraint during avian promoter evolution. This phenomenon might also be explained by the lower mutuality rate of AG motifs, as observed in primate genomes. However, the different mutability of dinucleotide repeats in the replication slippage model does not explain the higher rate of conserved TFBSs detected near ecSTRs with AG motifs. Therefore, we conclude that the divergent patterns of ecSTRs in avian lineages have been achieved by a combination of biological (e.g. effective population size), genetic (e.g. mutation rate), and structural (e.g. relative distance between motifs, stereospecific positioning) factors. Overall, our empirical approach has provided a broad perspective about avian promoter evolution. Such strategies are essential to obtain a comprehensive picture about the molecular evolution of vertebrate promoters.

## Methods

### Avian promoter sequences

Promoter sequences originating from five avian species [turkey (*Meleagris gallopavo*), duck (*Anas platyrhynchos*), zebra finch (*Taeniopygia guttata*), flycatcher (*Ficedula albicollis*), and chicken (*Gallus gallus*)] were used for the analyses. In this study, we defined the promoter region as 2 kb upstream from the TSS, because a previous study had indicated that the vast majority of reporter genes with 2 kb upstream region were able to drive expression in a dual luciferase assay^36^. Each sequence was obtained from the Ensembl BioMart database^37^ using homolog filters of either chicken genes (turkey, duck, zebra finch, and flycatcher) or turkey genes (chicken). In flycatcher sequences, we found long stretches of guanine or cytosine to fill contig gaps. Thus, 30 or longer uninterrupted stretches of guanine or cytosine were replaced with N to obtain a more precise estimation of GC% and the number of G4 motifs.

### Identification of sequence motifs

Phobos software ver. 3.3.12^38^ was downloaded and used to identify perfect and imperfect STRs in avian promoter sequences. For both STR types, we considered repeat unit sizes of 2 ~ 6 (i.e. dinucleotide, trinucleotide, tetranucleotide, pentanucleotide, and hexanucleotide) with at least 6 repeat units in perfect (pure) repeat or with 5.5 units in imperfect (interrupted) repeat. The following values were selected for an imperfect STR search: maximum number of successive Ns allowed in a repeat: 2; mismatch score: −5; indel score: −5; recursion depth: 5. All STRs were grouped into their canonical motifs for simplicity. We examined the frequencies of STR motifs to determine whether there was any statistical difference in their distributions between avian species.

### Screening for evolutionary conserved short tandem repeats

The gene ID converter (dbOrtho) implemented in the biological database network or BioDBnet^39^ was used to search for orthologous genes between chicken and other birds. Next, we investigated the ratio of STR motifs identified in chicken promoters that shared the same structural features with those of other birds. The avian promoter sequences were continuously screened out using following criteria: (1) promoter sequences in the chicken and other avian species were examined for whether they had any STR motif (di- to hexanucleotide repeats with at least 6 repeat units) in any promoter region of the orthologous gene (2) further screening was carried out to examine whether both orthologs would have the same STR motif regardless of the promoter site they were located in. (3) we finally examined whether the STR locations matched with those of chicken STRs. Orthologous STRs were considered to be in the same location of promoters if both STR loci were detected within ±200 bp relative to the positions in chicken STR sites. Therefore, in this study, we defined ecSTRs as the STR loci that had matched characteristics of gene symbol, repeat motif, and location in promoter regions between chicken and at least one of four other birds. Note that neither the number of repeat units nor repeat purity was considered in these screening procedures.

### Intrinsic features of evolutionary conserved loci within the avian promoters

The percentage of ecSTRs was calculated for each bird species, and compared among species, as well as between perfect and imperfect repeats. We excluded data derived from CG motifs in a dinucleotide repeat comparison due to extremely low frequency, while only AGG and CCG motifs were used in the comparison of trinucleotide repeats. When we compared the ratio of ecSTRs between promoter regions, the promoter sequence was divided into three regions: distal (−2000 ~ −501 bp), proximal (−500 ~ −101 bp), and core (−100 ~ −1 bp). We also examined the mutational bias toward the expansion and contraction of ecSTRs. The average number of repeat units in dinucleotide loci was calculated for each avian lineage. Next, we sorted ecSTRs into three categories (gain, loss, and unchanged) based on the number of repeat units relative to chicken orthologs. We examined the tendency of ecSTRs to gain or loss in the number of repeat units between loci with perfect or imperfect repeats.

### Distribution of TFBSs co-occurring with evolutionary conserved repeats

The CONREAL web server^40^ allowed us to identify TFBSs that were conserved between two orthologous promoter sequences by a phylogenetic footprint approach. We used relatively strict search parameters for the TFBS prediction: threshold for position weight matrix: 90%; length of flanks to calculate homology: 15 bp; threshold for homology: 75%. We selected only CONREAL prediction mode for global alignment. In this analysis, the JASPER^41^ database was chosen for reference TFBS profiles. Predicted TFBSs were sorted by location in the chicken promoter for each 100 bp bin to examine any bias in their distributions. The relative distance between ecSTR and TFBS was evaluated in each ecSTR locus. The data from the two passerines were combined due to their having a smaller number of ecSTRs than turkey and duck. To avoid counting TFBSs embedded within STR elements, TFBSs that had biased distributions in the vicinity of ecSTRs (±20 bp) were excluded from the data analyses.

## Additional Information

**How to cite this article**: Abe, H. and Gemmell, N. J. Evolutionary Footprints of Short Tandem Repeats in Avian Promoters. *Sci. Rep.* **6**, 19421; doi: 10.1038/srep19421 (2016).

## Supplementary Material

Supplementary Information

## Figures and Tables

**Table 1:** Summary of sequence motifs identified in the avian promoter.

	Chicken			Turkey			Duck			Zebra finch			Flycatcher		
Genome	*Galgal4*			*UMD2*			*BGI_duck_1.0*			*taeGut3.2.4*			*FicAlb_1.4*		
Ortholog/total	13754/17108			13664/1500			13583/16450			15633/18618			13554/16266		
GC %	50.43			45.71			45.72			47.21			48.71*		
STR	pfc	imp	Units	pfc	imp	Units	pfc	imp	Units	pfc	imp	Units	pfc	imp	Units
Dinucleotide	881	1843	7.64	670	1387	7.60	901	1481	7.26	870	2206	7.60	635	1510	8.91
Tri-	386	726	7.67	164	354	7.46	248	511	7.79	419	824	7.92	272	453	9.26
Tetra-	128	314	9.89	82	212	7.64	129	311	9.49	118	261	12.59	172	207	24.91
Penta-	146	284	10.75	49	138	8.19	82	178	12.82	155	325	12.21	176	178	31.47
Hexa-	24	91	9.81	8	31	7.25	14	69	13.01	69	168	11.10	122	114	32.18
Total STRs	1565	3258		973	2122		1374	2550		1631	3784		1377	2462	
Occupation (%)	0.4402			0.2615			0.4038			0.4593			0.7402		
G4	3963			1200			1817			3340			2952		
Occupation (%)	0.5106			0.1732			0.2643			0.3749			0.4580		

^*^Thirty or longer uninterrupted stretches of guanine or cytosine are replaced with N. Abbreviations are pfc [perfect (pure) repeat], imp [imperfect (interrupted) repeat], G4 (G-quadruplex).

**Table 2:** The number of conserved short tandem repeat loci with gain or loss of repeat units.

Motif	Unit gain				Unit loss				Unchanged			
	AC	AG	AT	Tri-*	AC	AG	AT	Tri-*	AC	AG	AT	Tri-*
Turkey	19 (0.28)	11 (0.19)	10 (0.30)	12 (0.33)	27 (0.39)	14 (0.25)	10 (0.30)	9 (0.25)	23 (0.33)	32 (0.56)	13 (0.39)	15 (0.42)
Duck	7 (0.33)	6 (0.24)	4 (0.36)	5 (0.38)	9 (0.43)	8 (0.32)	3 (0.27)	4 (0.31)	5 (0.24)	11 (0.44)	4 (0.36)	4 (0.31)
Zebra finch	8 (0.53)	9 (0.38)	3 (0.5)	**12 (0.5)**	3 (0.2)	12 (0.5)	1 (0.17)	**10 (0.42)**	4 (0.27)	3 (0.12)	2 (0.33)	2 (0.08)
Flycatcher	6 (0.38)	6 (0.33)	1 (0.33)	4 (0.57)	7 (0.44)	8 (0.44)	0 (−)	0 (−)	3 (0.19)	4 (0.22)	2 (0.67)	3 (0.43)

The number in brackets indicates the frequency of each STR category in each bird.

Numbers underlined and bold indicate significant levels of losses and gains, respectively (χ^2^ test; p < 0.05).

CG motifs are excluded from this table due to extremely low frequency.

^*^Trinucleotide repeats.
