# Supplementary material for: Evolutionary Footprints of Short Tandem Repeats in Avian Promoters
Source: Sci Rep. 2016 Jan 14;6:19421. doi: 10.1038/srep19421 (PMC4725869; doi:10.1038/srep19421)
Supplement: Supplementary Information [file srep19421-s1.pdf]

SUPPLEMENTALY INFORMATION

Evolutionary Footprints of Short Tandem Repeats in Avian Promoters

Hideaki Abe[1,*,†] and Neil J Gemmell[1,2]

[1] Department of Anatomy, University of Otago, Dunedin 9054, New Zealand
[2] Allan Wilson Centre for Molecular Ecology and Evolution, University of Otago, Dunedin 9054, New Zealand

[*] Author for Correspondence/ [†] Present address:
Hideaki Abe
Wildlife Research Center, Kyoto University, Sakyo, Kyoto 6068203, Japan
E-mail: habe2031@gmail.com

Legend to Supplementary Figures

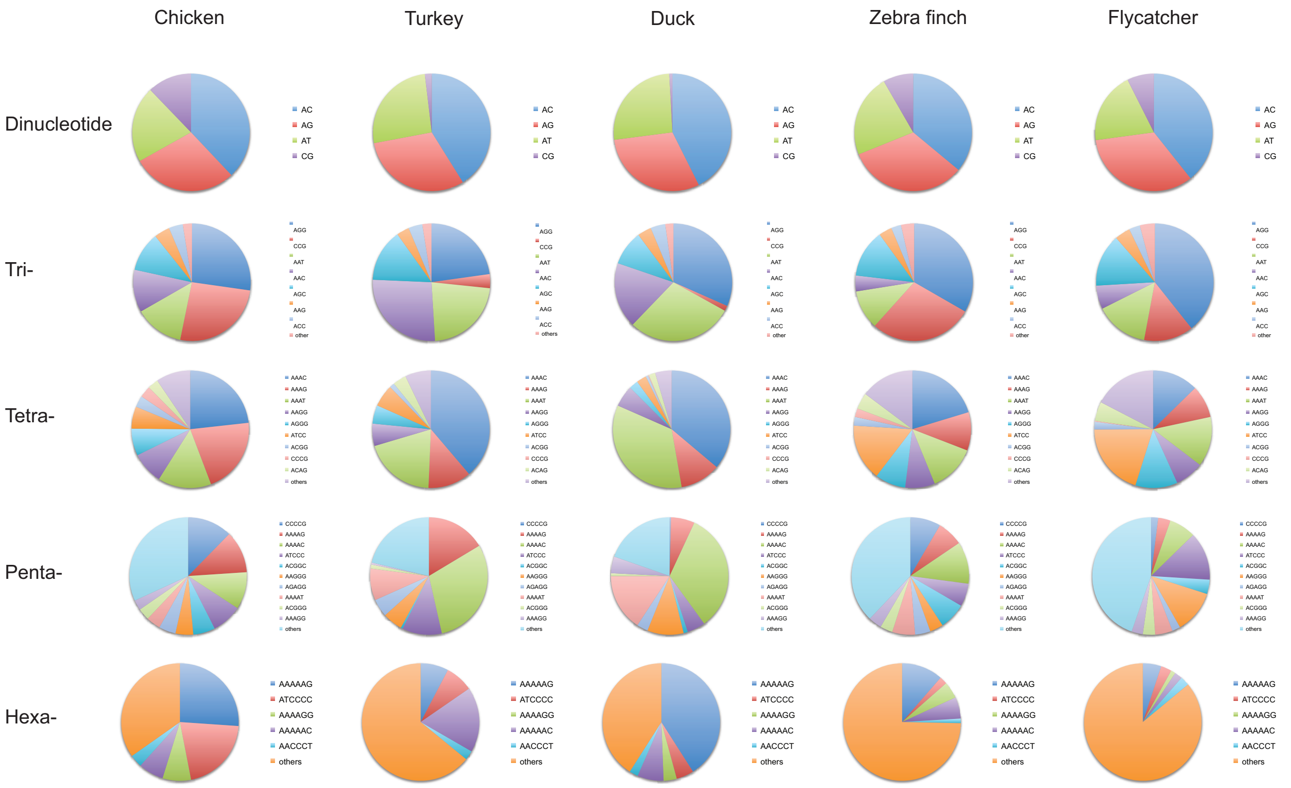

Figure S1. Motif frequency distribution for short tandem repeats (STRs) identified in avian promoters. In this study STRs were counted only if they contained at least 6 tandem repeat units in repetitive sequences. Data from perfect and imperfect repeats were combined here.

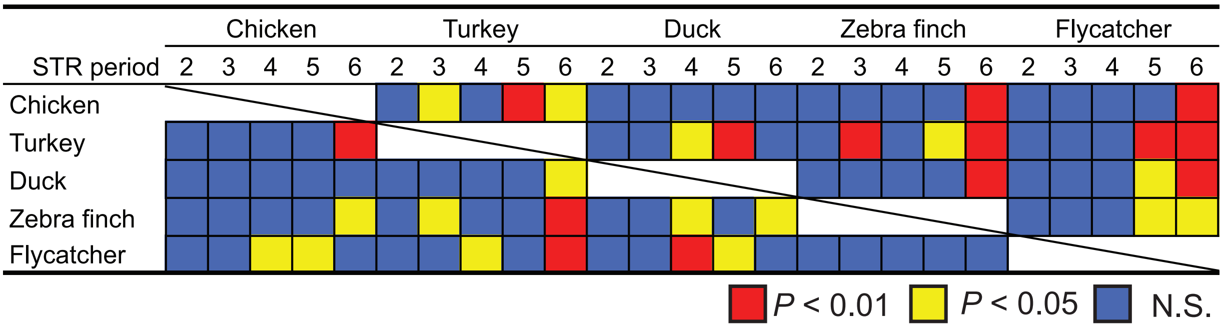

Figure S2. A pairwise comparison of motif frequencies among avian species. The F-test is used to identify a significantly biased distribution of STR motifs between bird taxa.

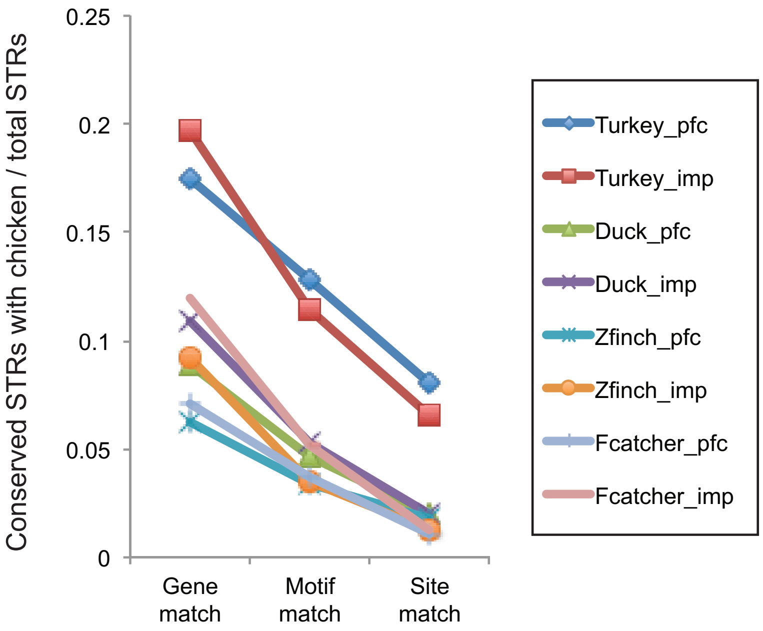

Figure S3. Screening for evolutionary conserved STRs among avian promoters based on the criteria of sequence motif profiles. See *Methods* section for more details about the screening criteria. Abbreviations used in this figure are pfc [perfect (pure) repeat], imp [imperfect (interrupted) repeat], Zfinch (zebra finch), and Fcatcher (flycatcher).

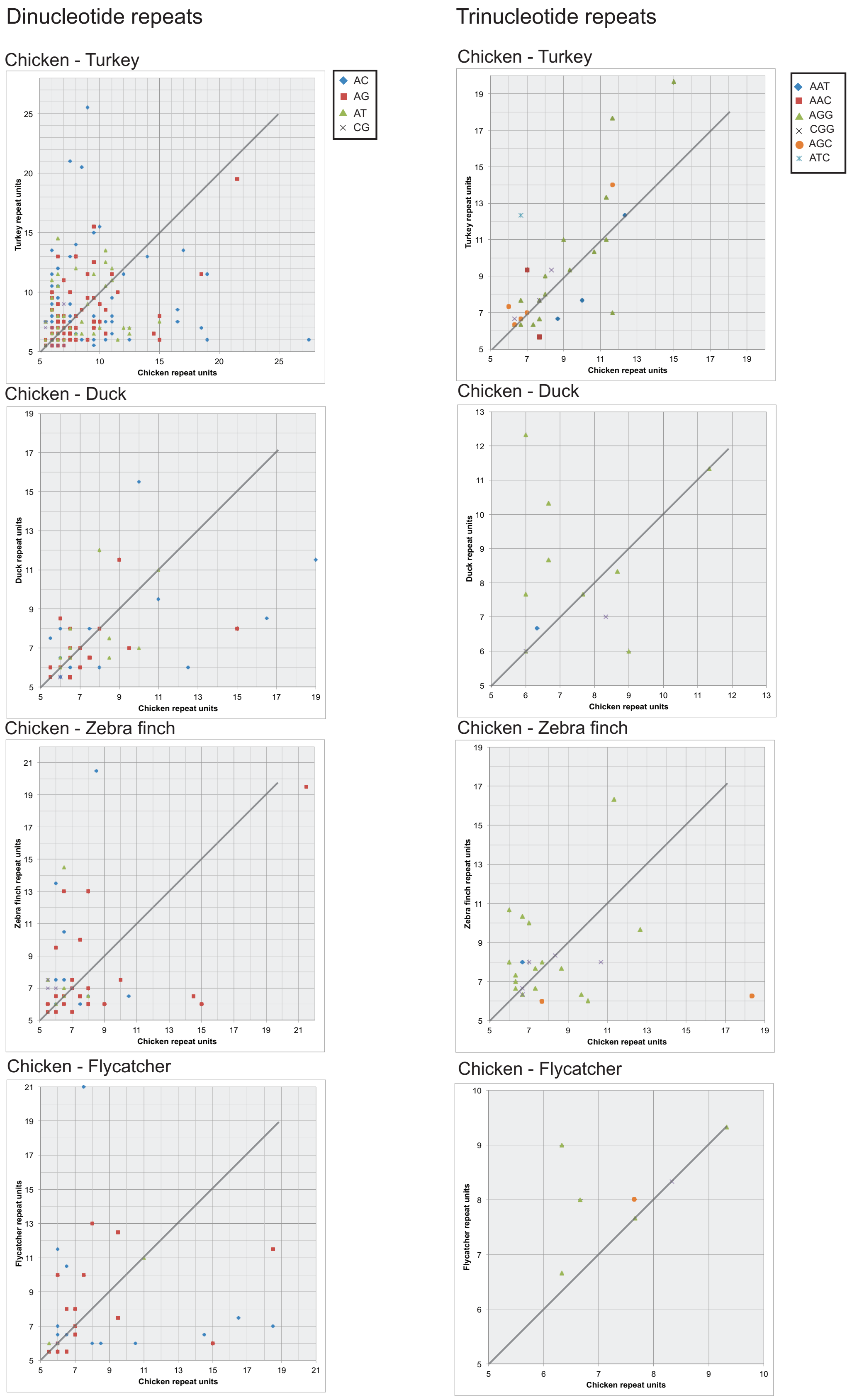

Figure S4. Trends in the number of repeat units embedded within evolutionary conserved STRs relative to those of chicken orthologs.

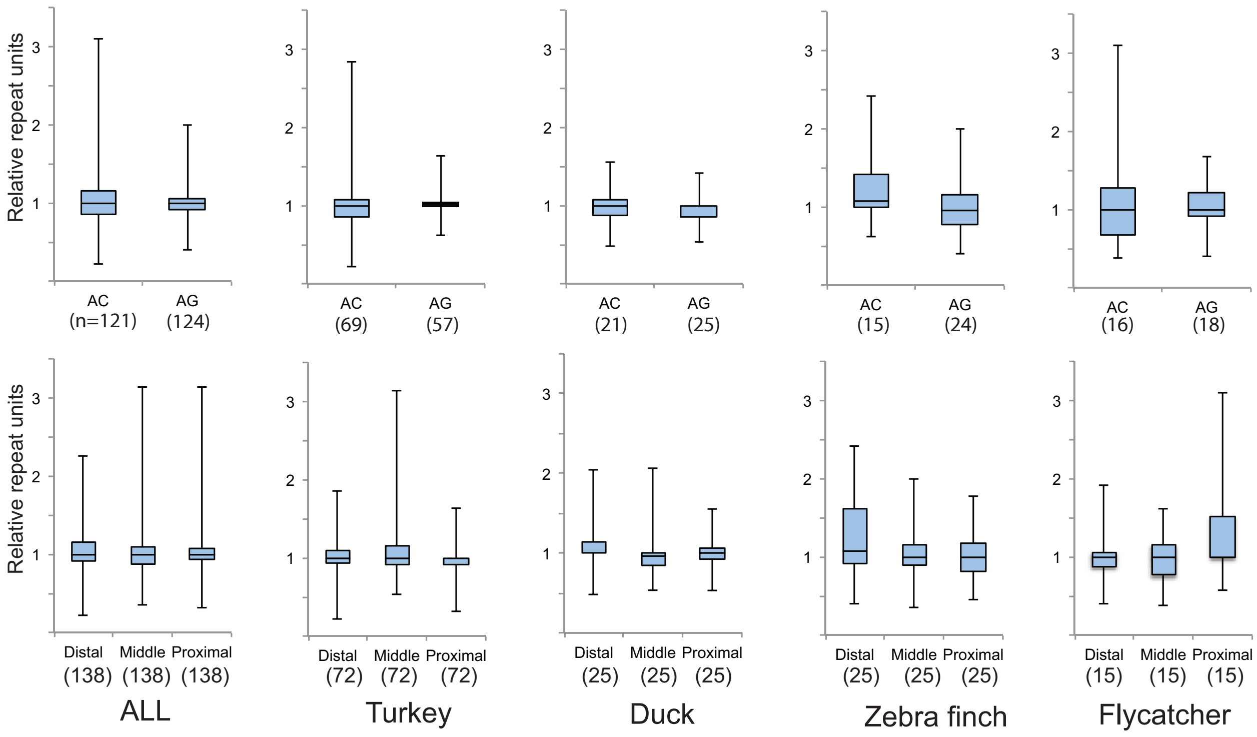

Figure S5. Repeat unit length of the evolutionary conserved STRs (ecSTRs) relative to chicken orthologs. Values in the parentheses indicate the number of ecSTRs loci analyzed here.

Chicken - Turkey

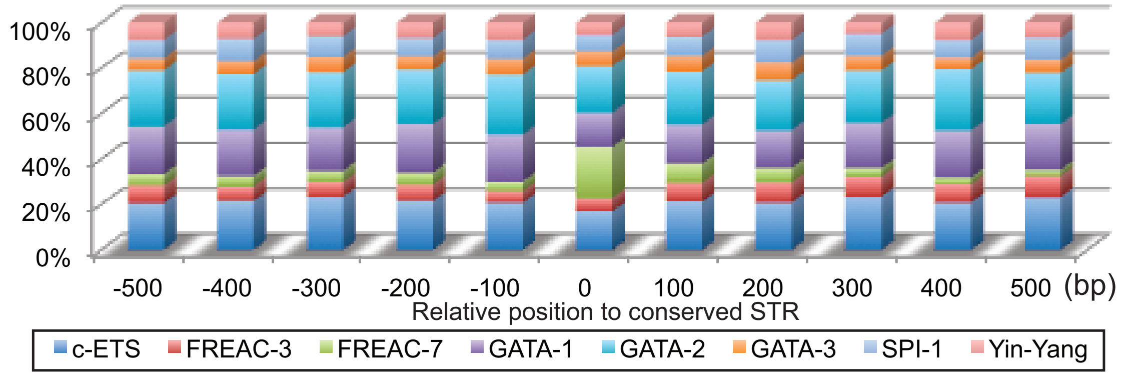

Chicken - Duck

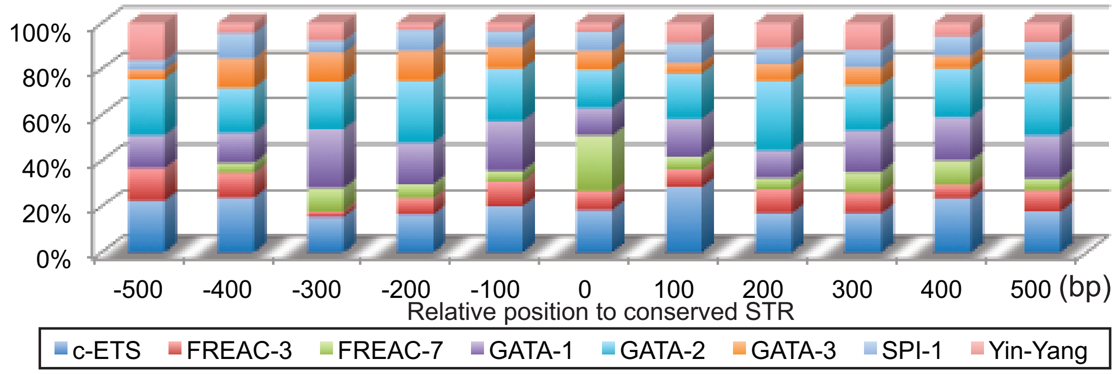

Chicken - Passerines

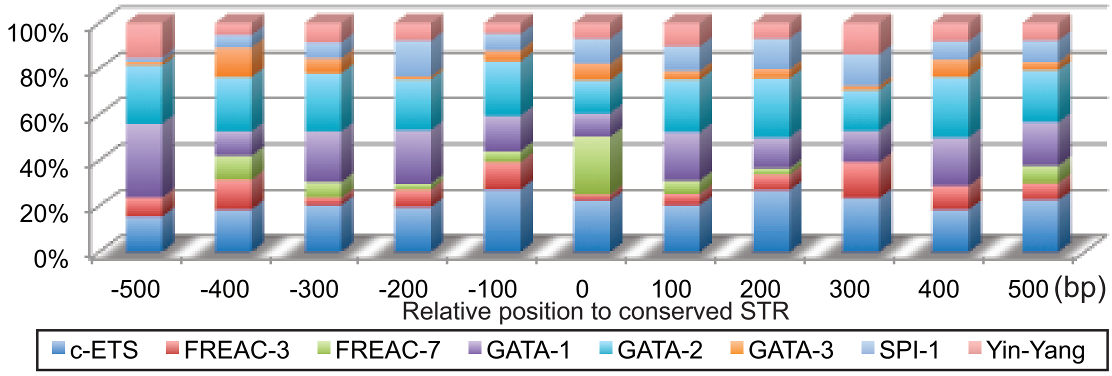

Figure S6. The relative distance of transcription factor binding site (TFBS) each from evolutionary conserved STRs identified between chicken and other bird taxa. The vertical axe of the stacked graph shows the percentage of TFBSs detected in each 100 bp bin.
